# The Beneficial Effects of Moxibustion on Overweight Adolescent Girls

**DOI:** 10.1155/2021/1943181

**Published:** 2021-05-10

**Authors:** Yuan-Chieh Yeh, Chin-Chang Chen, Ching-Yi Cheng, Hsin-Ning Chang, Tse-Hung Huang

**Affiliations:** ^1^Department of Traditional Chinese Medicine, Chang Gung Memorial Hospital, Keelung, Taiwan; ^2^Program in Molecular Medicine, School of Life Sciences, National Yang Ming University, Taipei, Taiwan; ^3^Department of Anatomy, School of Medicine, China Medical University, Taichung, Taiwan; ^4^Graduate Institute of Health Industry Technology, Research Center for Chinese Herbal Medicine and Research Center for Food and Cosmetic Safety, College of Human Ecology, Chang Gung University of Science and Technology, Taoyuan, Taiwan; ^5^Department of Pulmonary Infection and Immunology, Chang Gung Memorial Hospital at Linkou, Taoyuan, Taiwan; ^6^Graduate Institute of Clinical Medicine Sciences, College of Medicine, Chang Gung University, Taiwan; ^7^School of Nursing, National Taipei University of Nursing and Health Sciences, Taipei, Taiwan

## Abstract

Among adolescent girls, overweight or obesity has both physical and psychological involvement. We conducted a randomized controlled trial of moxibustion using a moxa burner. Fifty-four eligible girls aged 15–18 years with a body mass index (BMI) greater than 25.3 were enrolled in the study. The girls were randomly allocated to the treatment (*n* = 27) and control (*n* = 27) groups. The girls underwent treatment three times per week for 8 weeks (24 treatments). Moxibustion was applied to the RN12, RN6, ST25, ST36, and SP6 acupoints. Physical assessments were BMI, waist-to-hip ratio (WHR), and body fat ratio (BFR). Psychological outcomes were measured using the Rosenberg Self-Esteem Scale (RSE). Data were collected at the beginning of the study (baseline), week 4, and week 8. Of the 54 participants, 46 completed the trial. The difference in mean BMI from baseline between the two groups was 0.097 (*p*=0.655) at week 4 and −0.794 (*p*=0.001) at week 8. The mean WHR of the treatment group was significantly reduced compared with baseline, with a −0.011 (*p*=0.017) and −0.035 (*p* < 0.001) mean change at weeks 4 and 8, respectively. The mean BFR was slightly reduced (−0.253;*p*=0.474 ) at week 4 compared with baseline in the treatment group. At week 8, it was significantly reduced (−2.068; *p* < 0.001) from baseline in the treatment group. The mean RSE in the treatment group showed no significant increase from baseline at week 4 (0.155 points, *p*=0.803), but it improved significantly from baseline at week 8 (1.606 points, *p*=0.021) compared to that in the control group. No obvious adverse effect was reported during this study. Moxibustion using a moxa burner may be an effective and safe intervention for overweight adolescent girls, having both physical and psychological benefits.

## 1. Introduction

Overweight in adolescents is a major health concern worldwide because it is a strong predictor of high risks of cardiovascular disease, type 2 diabetes, and other health problems [1, 2]. Moreover, adolescents with overweight or obesity have a greater risk of adulthood overweight or obesity regardless of race or ethnicity [3]. Because the prevalence of overweight in adolescents has increased in several developed and developing countries [4–6], it has garnered more public attention to promote health improvements. In Taiwan, the prevalence of adolescent overweight was 12%–34% in a large-scale nationwide survey [7]. Being overweight or obese is more likely to contribute to major psychological problems among girls than among boys [8, 9]. Adolescent girls who developed a negative body image were found to be at a greater risk of subsequent psychological difficulties, such as frustration or a sense of failure [10]. Social aspects of overweight or obesity, such as prejudice and discrimination, also play a role in adolescent mental development as adolescents mature into adulthood.

Traditional Chinese medicine (TCM) techniques, including acupuncture, body or auricular acupoint stimulation, and acupoint catgut embedding, have become increasingly widely applied for controlling overweight and obesity. Moxibustion is a thermal stimulation method that employs ignited material applied onto or above the surface of the skin of a patient [11]. It can also be applied above acupoints on the surface of the patient's skin by using moxa sticks [12]. From the viewpoint of traditional Chinese medicine, moxibustion dredges meridians, relieves stagnation, and regulates qi-blood balance. This simple and safe therapeutic technique has been employed to treat myriad diseases [13] and is relatively noninvasive compared with acupuncture or acupoint catgut embedding. However, clinical trials on moxibustion and adolescents with overweight remain scant.

This clinical trial evaluated the effectiveness of moxibustion on adolescent girls with overweight. We hypothesized that, at the end of the 8-week intervention period, patients in the moxibustion group would exhibit more substantial improvement in both physical and psychological function than would those in the control group.

## 2. Materials and Methods

### 2.1. Subject Selection

A randomized controlled trial was executed to ascertain the effectiveness of moxibustion among adolescent girls with overweight who were from a nursing school in Northern Taiwan. In total, 54 eligible participants aged from 15 to 18 years were enrolled. The participants all had a body mass index (BMI) greater than 25.3, which is the World Health Organization's (WHO's) definition of overweight. Participants were randomly allocated to the treatment group (*N* = 27) or control group (*N* = 27) using computer-generated numbers. Participants who, during the trial, had catastrophic diseases, wounds on the abdomen, or any signs of acute inflammation, as well as those who were pregnant, were excluded. Moreover, participants who missed more than three consecutive sessions during the 8-week intervention were excluded from the trial. The process of this randomized controlled trial was approved by the Institutional Review Board of the Chang Gung Memorial Foundation (95–1478B).

### 2.2. Moxibustion Intervention

**Figure 1:**
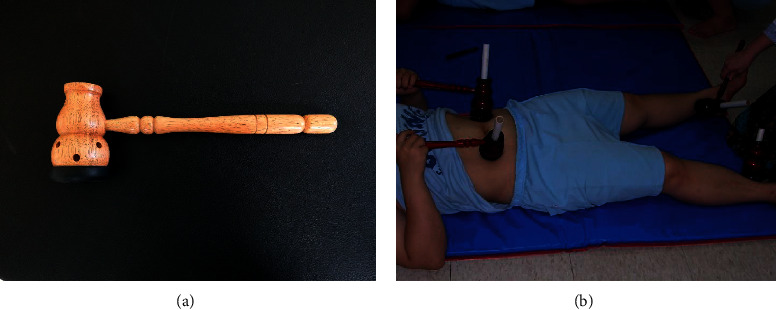
Moxa burner (a) and an example of moxibustion intervention (b). The participant (lay down) held two moxa burners applied to bilateral ST25, while the fellow participant held two burners applied to bilateral SP6 for the first 10 minutes. After the moxa burners were cleaned, the participant shifted two moxa burners to RN12 and RN6, while the fellow participant applied the moxa burners to bilateral ST36 for another 10 minutes. After a complete 20-minute intervention, the two participants swapped their positions for another 20-minute moxibustion treatment.

Moxibustion interventions were performed thrice weekly for 8 weeks (24 treatments in total). Moxibustion was applied to the RN12 (*zhong wan*), RN6 (*qi hai*), ST25 (*tian shu*), ST36 (*zu san li*), and SP6 (*san yin jiao*) acupoints. The intervention was performed using a moxa burner (Figure 1(a)), with placing one end of a moxa stick; therefore, the distance between the lit end of the moxa stick and the corresponding acupoint was stable. A metal mesh was attached to the bottom of the moxa burner to prevent burns resulting from falling ash. Each standardized moxa stick weighed 30–32 g. For moxibustion intervention of 10 min/acupoint, 4 ± 1 g of moxa material was ignited. After a brief introductory session of the usage of a moxa burner and the locations of all acupoints to ensure standardization of the treatment process, the participants were asked to work in pairs. One participant lay down and held two moxa burners applied to bilateral ST25, while another fellow participant held two burners applied to bilateral SP6 (Figure 1(b)) for the first 10 minutes. Then, the participants took out all moxa sticks to clean the ashes in the moxa burners, kept moxa sticks ignited, and inserted them into burners. Afterwards, the participant shifted two moxa burners to RN12 and RN6, while the fellow participant applied the moxa burners to bilateral ST36 for another 10 minutes. After a complete 20-minute moxibustion treatment, the participant and her partner exchanged their positions for another 20-minute intervention. During the entire 40-minute intervention, the participants could adjust the moxa sticks if they felt the skin too hot or not hot enough; otherwise, they were told to keep the moxa sticks in place to avoid experimental bias. Participants were told to maintain their daily routines following each intervention. Participants who missed one treatment were asked to perform moxibustion at home using moxa burners and material provided by the research staff.

### 2.3. Control Intervention

Participants in the control group were encouraged to maintain their daily routines during the 8-week study period. They were asked not to participate in moxibustion or acupuncture treatment or new exercise programs or diets. They received a 1-week (i.e., three treatments) supply of moxa burners and material following the study period and were taught how to use them, in return for their participation.

### 2.4. Assessment

The physical and psychological variables were measured at the beginning of the study (baseline), week 4, and week 8. The physical outcomes were changes in the BMI, waist-to-hip ratio (WHR), and body fat ratio (BFR). Data were acquired using a digital medical scale (HW-999, Super View, Taiwan) for BMI measurement and In Body 3.0 for WHR and BFR measurement in the laboratory to avoid measurement bias.

The Rosenberg Self-Esteem Scale (RSE) was used to assess the psychological changes in the participants during the intervention [14]. The RSE is a validated social survey questionnaire and has been widely used in studies on adolescents with obesity [15–17]. In total, 10 items are rated on a 4-point scale on the RSE; total scores range from 10 to 40. Heavier weights of adolescent girls are correlated with lower RSE scores [18].

### 2.5. Data Analysis

SPSS v.15 for Windows was used for data processing and analysis. A statistically significant change was defined as *p* < 0.05. Differences in general demographic information between the two groups were measured using the chi-squared test. An independent-sample *t*-test was performed to compare the BMI, WHR, BFR, and RSE score between the two groups at the beginning of the study. Moreover, generalized estimating equations (GEEs) were used to assess the improvements in the BMI, WHR, BFR, and RSE score at the end of week 4 and week 8.

## 3. Results

**Figure 2:**
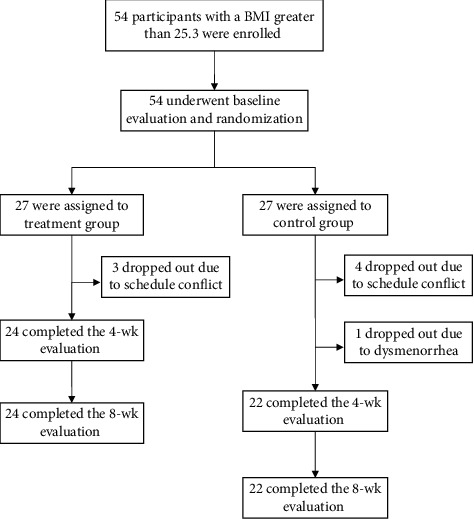
Screening, randomization, and evaluation algorithm.

In total, 54 participants were enrolled in this study.  Figure 2 shows the screening, randomization, and evaluation algorithm used in this study. Three participants in the treatment group and four participants in the control group dropped out of the study because of scheduling conflicts; one participant in the control group dropped out of the study because of dysmenorrhea at week 2. The dropout rate of this study was 14.9%.

### 3.1. Baseline Data of the Participants

Table 1 presents the baseline data of the participants after randomization, and no difference between the mean age, BMI, WHR, BFR, and RSE score was observed. The average participant was obese (mean BMI > 30), had high cardiovascular risk (mean WHR > 0.85), and had a high amount of body fat (mean BFR > 40). The mean RSE score was between 26 and 27.

Questionnaires on diet preference and medicine usage also revealed no differences (Table 2). Participants in the treatment group were more likely to have attempted to lose weight (*N* = 21, 87.5%) than were those in the control group (*N* = 14, 63.5%); however, this result was not statistically significant (*p*=0.058). Most participants did not take regular food supplements or medicine to lose weight. Heterogeneity was examined, and the result showed no statistical significance (*p* > 0.05).

### 3.2. Moxibustion Improved Both Physical and Psychological Outcomes of the Participants

**Figure 3:**
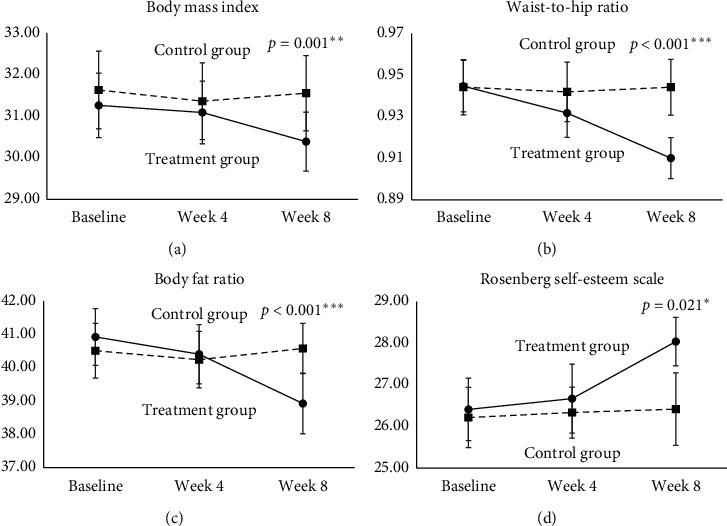
Mean changes in four variables at weeks 4 and 8 by the treatment group. The mean changes in body mass index (a), waist-to-hip ratio (b), body fat ratio (c), and rosenberg self-esteem scale (d) were measured at baseline, week 4, and week 8 between the treatment group and control croup. ^*∗*^*p* < 0.05, ^*∗∗*^*p* < 0.01, and ^*∗∗∗*^*p* < 0.001.

The physical and psychological evaluation data were analyzed using GEEs and are summarized in Table 3. Line charts of our data are provided in Figures 3(a)–3(d).

At week 4, the BMI of the treatment group was slightly increased compared with that of the control group (Table 3, Figure 3(a)). The difference in BMI change from baseline between the treatment group and control group was 0.097 (*p*=0.655) at week 4 and −0.794 (*p*=0.001) at week 8. In summary, after 8 weeks of moxibustion intervention, the treatment group participants' mean BMI score was 0.794 lower than that of the control group participants.

The treatment group exhibited a significant decrease (−0.011; *p*=0.017 and −0.035; *p* < 0.001) in WHR after 4 and 8 weeks' intervention, respectively, from baseline (Table 3, Figure 3(b)).

The treatment group showed a slight, nonsignificant decrease (−0.253; *p*=0.474) in BFR from baseline at week 4. However, at week 8, a significant decrease (−2.068; *p* < 0.001) in BFR from baseline was observed (Table 3, Figure 3(c)).

The RSE score of the treatment group was slightly higher (0.155; *p*=0.803) at week 4. At week 8, the treatment group showed a significant improvement from baseline (1.606 points; *p*=0.021) than the control group (Table 3, Figure 3(d)).

### 3.3. Moxibustion Caused No Adverse Effects during the Trial

No obvious adverse effects, such as burn injuries or irritation, were observed during the 8-week intervention. One participant dropped out of the control group because of dysmenorrhea at week 2; this was unrelated to the moxibustion intervention.

## 4. Discussion

To the best of our knowledge, this is the first integrated clinical trial to evaluate the effects of moxibustion on adolescent girls with overweight. Moxibustion is usually applied in combination with other acupuncture treatments, such as needle acupuncture, electroacupuncture, laser acupuncture, or even more invasive acupoint catgut embedding therapy [19, 20]. On the aspect of weight reduction, moxibustion with warming needle acupuncture may be one of the optimal methods in losing weight [20]. However, there was no clinical trial investigating purely moxibustion in losing weight. Among 34 eligible trials analyzed in one literature review, only one study used moxibustion with other acupuncture therapies to lose weight [20]. Herein, we conducted a randomized controlled trial to apply simply moxibustion in treating obesity or overweight. Our results support moxibustion's efficacy in reducing the BMI, WHR, and BFR, as well as increasing self-esteem in adolescent girls with overweight. All participants tolerated moxibustion using the noninvasive and easy-to-use moxa burner favorably without severe adverse events. The findings of this study may have important implications for managing the global health issue—adolescent obesity or overweight.

We enrolled all participants according to the WHO definition of overweight (i.e., a BMI greater than 25.3). However, the average BMI in our study participants was more than 30 in both groups (Table 1), which is defined as obese.

The results of our questionnaires revealed that 35 (76.08%) of the participants had previously attempted to lose weight (Table 2). Most adolescents with overweight or obesity reported that they had attempted to lost weight, according to an international survey [21]. Furthermore, most respondents to the survey in our study stated that they did not spend money on losing weight, which may have been because of economic status. Additionally, more than 90% of participants in one study reported a habit of late-night snacking, which is a risk factor for adolescent obesity [22]. Another study indicated that circadian rhythm and food intake interact to play a pivotal role in the development of adolescent obesity, perhaps because of the strong adverse association between glucose and insulin at nighttime [23].

According to the viewpoint of traditional Chinese medicine, the disease pattern observed in obese adolescents was yang deficiency and qi stagnation. Moxibustion has been used for warming yang and dredging meridians, which is favorably indicated for treating obesity. In addition to moxibustion, acupuncture and acupoint catgut embedding are used to treat adolescents with obesity in Taiwan. However, several adverse effects of these treatments—such as pain, hematoma, and granuloma—have been reported [24]. The safe and easy-to-use moxa burner and material used in our study may have contributed to the low withdrawal rate (14.8%) compared with that in studies using needle acupuncture (25%–27%) [25].

Our results revealed that moxibustion reduced the WHR earlier than the other measures, with a significant reduction (*p*=0.017) in WHR observed at week 4 (Table 3, Figure 3(b)). This anthropometrical change may reduce the risk of cardiovascular disease because WHR is strongly associated with coronary artery calcification in young adults, and this parameter is frequently used for cardiovascular risk evaluation [26]. This is consistent with the correlation between visceral fat and coronary atherosclerosis. The body shape of the participants was more “apple-shaped” (WHR > 0.85) at baseline and seemed to be more “pear-shaped” following the moxibustion intervention. The results revealed that moxibustion reduces the cardiovascular risk of girls with overweight or obesity.

Our findings were broadly consistent with those of other studies on acupuncture or moxibustion [19, 27]. The results may be because of the abdominal acupoints used in our study: RN12, RN6, and bilateral ST25 are traditionally used for adjusting bowel movements and treating constipation, whereas ST36 and SP6 are used for dredging meridians and relieving stagnation, especially in the stomach and spleen channels, with stagnation also correlated with obesity. One clinical trial indicated that warming needle acupuncture applied to abdominal acupoints (RN12, RN9, RN6, RN3, ST25, and ST28) may have long-term therapeutic effect on simple obesity with spleen deficiency pattern [28]. Previous study indicated that moxibustion-like thermal stimulation to the mouse abdomen decreased the size of white adipose tissue and induced formation of beige adipocytes [29]. Electroacupuncture stimulation to acupoints RN12, SP6, and ST36 may regulate gastrointestinal motility *in vivo* through the vagus-gastric neural pathway [30, 31]. Similarly, diet-induced obese rats treated by electroacupuncture applied to ST25, RN12, SP6, and ST36 showed the mechanisms to reduce weight and appetite may be related to hypothalamic Tsc1 promoter demethylation and mTORC1 signaling pathway inhibition [32]. However, the detailed molecular mechanism responsible for weight reduction by simply moxibustion is yet to be clearly identified.

Other physical variables, such as BMI and BFR, showed no significant decrease until week 8. These results were similar to those of other studies that used acupuncture to treat children with obesity [33], suggesting that acupuncture significantly reduces visceral fat and WHR without significantly changing body weight.

The association between overweight or obesity and self-esteem was reported to be tenuous in other studies on Han and other ethnic groups [9, 34, 35]. However, overweight or obesity may increase the risk of developing body image dissatisfaction, which may in turn impair self-esteem [36]. In particular, girls with overweight in our study reported an increase in self-esteem after moxibustion (Table 3, Figure 3(d)). The mean baseline RSE scores of the two groups were 26.58 (treatment) and 26.36 (control), which did not statistically differ. As expected, moxibustion successfully increased the mean RSE score by 1.606 in the treatment group, indicating that the self-esteem of the participants was increased by the intervention.

The moxa burner is a relatively safe device, according to our study. The burner, which has a fine metal mesh at the bottom, allows heat to pass without ashes falling onto the skin. During the 8-week intervention, no adverse effects or burns were reported, probably because this safe moxibustion device was used and its method of use was effectively explained. Although other studies have reported potentially adverse effects from moxibustion, such as allergies, burns, and infections [37], no participant in our treatment group withdrew because of these adverse effects.

### 4.1. Limitations

Our study had several limitations. First, the trial was not double blinded because performing sham moxibustion is difficult. To date, no validated double-blinded studies on moxibustion have been reported in the literature. Other studies have performed sham moxibustion by using devices to prevent heat radiating from the moxa burner to the patient's skin [38]. However, the sensation of heat may play a role in the process of moxibustion. Second, this study was conducted in only one nursing school in Northern Taiwan. Thus, the generalization of the results to other populations with different backgrounds may be limited. Third, the measurements were taken thrice only, without long-term follow-up, because of our limited research funding and resources. Fourth, we may enroll more eligible samples in our further study to strengthen our results because the error bars in Figure 3 seem to be excessive. We believe the results of our study may provide a comprehensive knowing in designing a larger-scale randomized controlled trial in the future to improve statistical power.

## 5. Conclusions

Moxibustion intervention may help improve some physical and psychological variables of adolescent girls with overweight, including the WHR and BFR, as well as the BMI and RSE score. This simple and safe therapeutic method is worthy of promotion by public health authorities.

## Figures and Tables

**Table 1:** Baseline characteristics of the participants.

Variable	Treatment group (*N* = 24)	Control group (*N* = 22)	*p*
	Mean ± SD	Mean ± SD	
Age, yr	17.17 ± 0.92	17.50 ± 1.01	0.247
Body mass index	31.26 ± 3.80	31.63 ± 4.39	0.761
Waist-to-hip ratio	0.94 ± 0.06	0.94 ± 0.62	0.978
Body fat ratio	40.93 ± 4.18	40.51 ± 3.86	0.728
RSE^*∗*^	26.58 ± 4.13	26.36 ± 3.82	0.853

^*∗*^RSE: Rosenberg Self-Esteem Scale.

**Table 2:** Results of the questionnaire on diet habits and medicine use of the participants.

Variable	Treatment group (*N* = 24)	Control group (*N* = 22)	*p*
	Number (%)	Number (%)	
Ever attempted to lose weight			0.058
No	3 (12.5)	8 (36.4)	
Yes	21 (87.5)	14 (63.6)	
Exercised to lose weight			0.253
No	7 (29.2)	10 (45.5)	
Yes	17 (70.8)	12 (54.5)	
On diet to lose weight			0.253
No	7 (29.2)	10 (45.5)	
Yes	17 (70.8)	12 (54.5)	
Use of food supplement to lose weight			0.187
No	20 (83.3)	21 (95.5)	
Yes	4 (16.7)	1 (4.5)	
Use of herbal medicine to lose weight			0.268
No	19 (79.2)	20 (90.9)	
Yes	5 (20.8)	2 (9.1)	
Use of other medicine to lose weight			0.333
No	23 (95.8)	22 (100)	
Yes	1 (4.2)	0 (0)	
Cost per month to lose weight			0.229
0 TWD	16 (66.7)	19 (86.4)	
1–1000 TWD	4 (16.7)	3 (13.6)	
1001–2000 TWD	1 (4.2)	0 (0)	
>2001 TWD	3 (12.5)	0 (0)	
A habit of a midnight snack			0.642
No	2 (8.3)	2 (9.1)	
Yes	22 (91.7)	20 (90.9)	
Prefer Chinese eating style			0.243
No	5 (20.8)	8 (36.4)	
Yes	19 (79.2)	14 (63.6)	
Prefer Western eating style			0.136
No	10 (41.7)	14 (63.6)	
Yes	14 (58.3)	8 (36.4)	

**Table 3:** Generalized estimating equation analysis of outcome measurements.

(A) body mass index	*ß*	Standard error	*p*
Group			
Treatment vs. control	31.627		
Time course			
4^th^ week vs. baseline	−0.268		
8^th^ week vs. baseline	−0.077		
Group ^*∗*^ time course (treatment vs. control)			
4^th^ week vs. baseline	0.097	0.218	0.655
8^th^ week vs. baseline	−0.794	0.248	0.001^*∗∗*^

(B) waist-to-hip ratio			
Group			
Treatment vs. control	0.944		
Time course			
4^th^ week vs. baseline	−0.002		
8^th^ week vs. baseline	−4.63		
Group ^*∗*^ time course (treatment vs. control)			
4^th^ week vs. baseline	−0.011	0.004	0.017^*∗*^
8^th^ week vs. baseline	−0.035	0.007	<0.001^*∗∗∗*^

(C) body fat ratio			
Group			
Treatment vs. control	40.509		
Time course			
4^th^ week vs. baseline	−0.268		
8^th^ week vs. baseline	0.068		
Group ^*∗*^ time course (treatment vs. control)			
4^th^ week vs. baseline	−0.253	0.353	0.474
8^th^ week vs. baseline	−2.068	0.548	<0.001^*∗∗∗*^

(D) Rosenberg self-Esteem Scale			
Group			
Treatment vs. control	26.364		
Time course			
4^th^ week vs. baseline	0.136		
8^th^ week vs. baseline	0.227		
Group ^*∗*^ time course (treatment vs. control)			
4^th^ week vs. baseline	0.155	0.622	0.803
8^th^ week vs. baseline	1.606	0.698	0.021^*∗*^

The outcome measurements are body mass index (A), waist-to-hip ratio (B), body fat ratio (C), and Rosenberg Self-Esteem Scale (*D*) (*N* = 46). ^*∗*^*p* < 0.05, ^*∗∗*^*p* < 0.01, ^*∗∗∗*^*p* < 0.001.

## Data Availability

The data supporting the findings of this study are available from the corresponding author upon request.

## Abbreviations

TCM: Traditional Chinese medicine
BMI: Body mass index
WHR: Waist-to-hip ratio
BFR: Body fat ratio
RSE: Rosenberg Self-Esteem Scale.
